# Supplementary material for: The genome sequence of the V-Pug moth, Chloroclystis v-ata (Haworth, 1809)
Source: Wellcome Open Res. 2025 Apr 22;10:197. [Version 1] doi: 10.12688/wellcomeopenres.23965.1 (PMC12332475; doi:10.12688/wellcomeopenres.23965.1)
Supplement: Peer review history

# Reviewer report: Reviewer response for version 1

**DOI:** 10.21956/wellcomeopenres.26441.r127001

**Published:** 2025-08-08

**Authors:** Laurence Despres

**Affiliations:** 1 Laboratoire d'Ecologie Alpine, Universite Grenoble Alpes, Auvergne-Rhône-Alpes, France

The authors report on the chromosome-level genome assembly of a female specimen of the V_Pug Chloroclystis v-ata (family Geometridae) *,* a common Palaearctic moth.

 The final assembly has a total length of 275.35 Mb and includes 17 chromosomes: 15 autosomes and the two sex chromosomes Z and W. The mitogenome was also assembled. The quality of the assembly was further assessed by the proportion of complete BUSCO genes recovered, which is very high (>98% of Lepidoptera database BUSCO genes).

 The genome assembly is of good quality and was constructed using appropriate methods : Pac Bio HiFi long reads (40-fold coverage) and Hi-C data to confirm scaffolding, following the high standard of the Darwin Tree of Life Project for results presentation.

Are sufficient details of methods and materials provided to allow replication by others?

Yes

Is the rationale for creating the dataset(s) clearly described?

Yes

Are the datasets clearly presented in a useable and accessible format?

Yes

Are the protocols appropriate and is the work technically sound?

Yes

Reviewer Expertise:

Evolutionary biology

I confirm that I have read this submission and believe that I have an appropriate level of expertise to confirm that it is of an acceptable scientific standard.

# Reviewer report: Reviewer response for version 1

**DOI:** 10.21956/wellcomeopenres.26441.r123283

**Published:** 2025-06-03

**Authors:** Bin Zhang

**Affiliations:** 1 Qingdao Agricultural University, Shenzhen, China

This genome note presents a high-quality chromosome-level genome assembly of *Chloroclystis v-ata* (V-Pug moth), an ecologically relevant species in the family Geometridae. The assembly statistics, sequencing depth, and completeness metrics (e.g., BUSCO score of 98.0%) are impressive and meet the standards of the Darwin Tree of Life project. The methodological transparency and use of standard pipelines (e.g., Hifiasm, BUSCO, MitoHiFi, HiGlass) are commendable.

 1. The manuscript would benefit from a brief discussion on how this genome resource may be used in future ecological or evolutionary studies of C. v-ata or related taxa.

 2. If feasible, providing a comparison with other Geometridae genomes (if available) would enhance the contextual value of this genome.

 3. While the figures are technically comprehensive, adding brief biological context (e.g., why Hi-C contact map matters) would help non-specialist readers.

Are sufficient details of methods and materials provided to allow replication by others?

Yes

Is the rationale for creating the dataset(s) clearly described?

Yes

Are the datasets clearly presented in a useable and accessible format?

Yes

Are the protocols appropriate and is the work technically sound?

Yes

Reviewer Expertise:

Enomology, Bioinformation.

I confirm that I have read this submission and believe that I have an appropriate level of expertise to confirm that it is of an acceptable scientific standard.
